# Substantial health and economic burden of COVID-19 during the year after acute illness among US adults at high risk of severe COVID-19

**DOI:** 10.1186/s12916-023-03234-6

**Published:** 2024-02-01

**Authors:** Amie Scott, Wajeeha Ansari, Farid Khan, Richard Chambers, Michael Benigno, Manuela Di Fusco, Leah McGrath, Deepa Malhotra, Florin Draica, Jennifer Nguyen, Joanna Atkinson, Jessica E. Atwell

**Affiliations:** 1 Global Real World Evidence, Pfizer Inc, New York, NY USA; 2 Global HEOR, Pfizer Inc, New York, NY USA; 3 Vaccines Medical Development & Scientific Clinical Affairs, Pfizer Inc, New York, NY USA; 4 Global Product Development Statistics, Pfizer Inc, New York, NY USA; 5 Medical Affairs, Pfizer Inc, New York, NY USA; 6 Medical Affairs, Pfizer Ltd, Tadworth, Surrey UK

**Keywords:** COVID-19, SARS-CoV-2, Long COVID, PASC, Post-COVID conditions

## Abstract

**Background:**

Post-COVID conditions encompass a range of long-term symptoms after SARS-CoV-2 infection. The potential clinical and economic burden in the United States is unclear. We evaluated diagnoses, medications, healthcare use, and medical costs before and after acute COVID-19 illness in US patients at high risk of severe COVID-19.

**Methods:**

Eligible adults were diagnosed with COVID-19 from April 1 to May 31, 2020, had ≥ 1 condition placing them at risk of severe COVID-19, and were enrolled in Optum’s de-identified Clinformatics^®^ Data Mart Database for ≥ 12 months before and ≥ 13 months after COVID-19 diagnosis. Percentages of diagnoses, medications, resource use, and costs were calculated during baseline (12 months preceding diagnosis) and the post-acute phase (12 months after the 30-day acute phase of COVID-19). Data were stratified by age and COVID-19 severity.

**Results:**

The cohort included 19,558 patients (aged 18–64 y, *n* = 9381; aged ≥ 65 y, *n* = 10,177). Compared with baseline, patients during the post-acute phase had increased percentages of blood disorders (16.3%), nervous system disorders (11.1%), and mental and behavioral disorders (7.7%), along with increases in related prescriptions. Overall, there were substantial increases in inpatient and outpatient healthcare utilization, along with a 23.0% increase in medical costs. Changes were greatest among older patients and those admitted to the intensive care unit for acute COVID-19 but were also observed in younger patients and those who did not require COVID-19 hospitalization.

**Conclusions:**

There is a significant clinical and economic burden of post-COVID conditions among US individuals at high risk for severe COVID-19.

**Supplementary Information:**

The online version contains supplementary material available at 10.1186/s12916-023-03234-6.

## Background

Post-COVID conditions are characterized by symptoms of COVID-19 that extend beyond the initial recovery from acute illness [1–3]. Presentation is highly variable across patients and may include residual symptoms that persist after acute illness, persistent organ dysfunction, or new symptoms or syndromes that develop after initial recovery from COVID-19 [1–3]. Post-COVID conditions often affect multiple organ systems, persist for several months, and have a substantial impact on daily functioning and productivity [4]. The clinical diagnosis, termed post-acute sequelae of COVID-19 (PASC), was officially defined and assigned an *International Classification of Diseases, Tenth Revision, Clinical Modification* (ICD-10-CM), code (U09.9) in October 2021 [5, 6], but a widely accepted case definition and associated symptom timeframe is still under development.

Multiple studies have identified post-COVID conditions as a frequent result of COVID-19 illness [1, 7–10], but prevalence estimates vary. The Centers for Disease Control and Prevention (CDC) recently reported that 38.2% of patients diagnosed with COVID-19 experienced ≥ 1 post-COVID condition from 30 to 365 days following diagnosis [10]. The World Health Organization reports a range from 10% to 20% of patients diagnosed with COVID-19 [2], and other studies have reported rates of up to 57% 6 months after diagnosis [8, 11–14]. Heterogeneous cohorts likely contribute to varying results given that certain characteristics, such as female sex and older age, are associated with developing long-term sequelae [15, 16].

Although the risk of post-COVID conditions has been associated with more severe cases of acute illness, it can occur across all levels of COVID-19 severity [1, 15, 17]. In a systematic review of > 2000 studies conducted worldwide among patients with varying disease severity, more than half of patients who recovered from acute illness experienced long-term sequelae for ≥ 6 months after diagnosis [8]. In 2 studies from Germany and the Faroe Islands of mainly nonhospitalized patients with asymptomatic or mild to moderate acute illness, approximately 28% to 53% of patients experienced persistent symptoms of COVID-19 months later [11, 13].

The potential for post-COVID conditions to develop in patients with mild or moderate illness is particularly relevant as new variants emerge, such as the highly transmissible Omicron variant, which is associated with milder illness compared with earlier strains [18–21]. In the context of these variants, immunity from natural infection or vaccination may provide better protection against severe disease than against overall infection [21]. Moreover, evidence suggests that risk of developing long-term symptoms may vary by circulating strain [22, 23]. The effects of these changes on population-level incidence of post-COVID conditions remain to be determined, but it is likely that the true burden of COVID-19 in terms of reduced quality of life, loss of productivity, strains on healthcare systems, and economic impact reaches far beyond acute infection.

Emerging data on long-term COVID-19 impact indicate a substantial healthcare burden. In 2 studies using the US Department of Veterans Affairs database, patients with COVID-19 compared with a control cohort had increased healthcare resource and medication use, as well as abnormalities across multiple organ systems, during the year after diagnosis [24, 25]. Other studies in patients with COVID-19 have identified increases in COVID-19**─**related healthcare provider visits, emergency department (ED) visits, and readmissions for up to 7 months after diagnosis [26–28]. However, comparison studies are often limited by the small numbers of patients and high variability between patient characteristics.

The aim of this retrospective analysis was to describe morbidity, healthcare resource use, and costs associated with the post-acute phase of COVID-19 among patients with underlying medical conditions or characteristics associated with higher risk of severe COVID-19 (hereafter referred to as “high risk”) in the United States [29]. This population was selected based on immediate relevance of the data to emerging COVID-19 treatments, which are authorized first for high-risk patients. To maintain focus on descriptive results and the guiding of hypothesis generation for future research, no formal comparisons were planned.

## Methods

### Study design and data source

This was a descriptive, retrospective, cohort study in patients diagnosed with COVID-19 between April 1 and May 31, 2020, in which each patient served as their own control for evaluation of diagnoses, medications, healthcare utilization, and costs before versus after acute COVID-19. Patients were identified using administrative health claims from Optum’s de-identified Clinformatics® Data Mart Database (CDM), which contains de-identified patient-level information derived from administrative healthcare claims from commercial and Medicare Advantage health plan members in all 50 states. Claims encompass medical and pharmacy healthcare services and include information regarding healthcare costs and resource utilization.

**Fig. 1:**
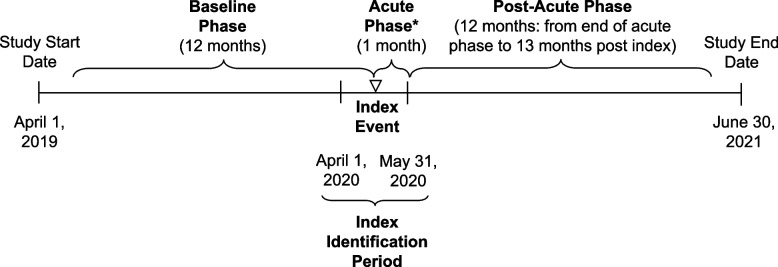
Study design. The date of first COVID-19 diagnosis was defined as the index date, and the index period was April 1 through May 31, 2020. For each patient, the baseline phase included the 12 months before the index date, and the post-acute phase included the 12 months after the end of the acute phase of COVID-19 illness. ^a^The acute phase, defined as the 30-day period after the index date, was excluded from analysis

The index period was April 1 to May 31, 2020, and the index date was defined as the date of the first COVID-19 diagnosis. Individual patient data were described during the 12 months before the index date (baseline phase) and during the 12 months after the end of the 30-day acute phase [3] of COVID-19 illness (Fig. 1). For patients whose hospital stays spanned across study phases, total numbers of events and associated costs were calculated per hospitalization day and were attributed to each phase based on the number of days falling within that phase.

### Participants

Patients were eligible for inclusion if they had ≥ 1 ICD-10 diagnosis code for confirmed COVID-19 (U07.1) during the index period. Patients were required to have continuous enrollment (gaps of ≤ 45 days were permitted) in Optum CDM over the 12 months before and 13 months after COVID-19 diagnosis, to be aged ≥ 18 years on the index date, and to have ≥ 1 high risk condition per CDC definition as of October 14, 2021 [29]. Sentinel code lists [30] were used to define these conditions when available, and all codes were reviewed by sponsor personnel with medical expertise (FD, JCA, NB, MLNFV). Criteria for immunocompromised individuals were developed from a recent literature review [31]. Inclusion criteria specified having a diagnosis code (ICD-10-CM), procedure code (ICD-10-Procedure Coding System [ICD-10-PCS], Current Procedural Terminology [CPT®], Healthcare Common Procedures Coding System [HCPCS]), or National Drug Code (NDC) for ≥ 1 of the listed conditions within the 12 months before the index date or being aged ≥ 65 years at the index date. Patients were excluded if they were hospitalized for ≥ 5 consecutive days during the baseline phase; spent any time at a long-term care facility, skilled nursing facility, inpatient rehabilitation, or hospice during baseline or at index date; had an ICD-10 code for confirmed COVID-19 before the index period; or died during the acute phase of COVID-19.

### Descriptive analysis

Outcomes of interest included ICD-10 diagnosis codes (other than confirmed COVID-19); medication use; outpatient visits and laboratory tests; ED visits; inpatient hospitalizations, including length of stay (LOS), intensive care unit (ICU) visits/LOS, ventilator use, and 30-day readmissions; and both standard and nonzero healthcare-associated costs. Standard costs were calculated based on all patients with a related visit or service, and nonzero costs were calculated based on all patients with a cost > 0 associated with that visit or service. The top 500 individual diagnosis codes were aggregated to their chapter and broader diagnostic categories and used for further analysis. Medications were analyzed via AnalySource® (Fayetteville, NY, USA) according to the Uniform System of Classification class. Diagnoses and medications were calculated as dichotomous occurrences during the 12 months before the index date (baseline phase) and the 12 months after the 30-day acute phase (post-acute phase); those with a prevalence of < 2% within the overall population during the baseline phase were excluded. Biologics were also excluded because the category primarily consisted of incompletely captured vaccine data. No adjustments were made for patients who died during the 12-month post-acute phase; all deaths that occurred during the post-acute phase were accounted for and reported. Continuous measures were totaled for each period. For each outcome, absolute and relative change from baseline to the post-acute phase was calculated using frequency counts.

Because older age is a risk factor for both increased COVID-19 severity and post-COVID conditions [21], data were separated into categories of patients aged 18 to 64 years or aged ≥ 65 years at the index date. To better understand the relationship between post-COVID conditions and acute COVID-19 severity, data were also stratified according to patient disposition during acute COVID-19 illness: not hospitalized, hospitalized without ICU admission, or admitted to the ICU. All variables were presented descriptively using mean ± SD or median (quartile 1 [Q1]; quartile 3 [Q3]) for continuous variables and frequencies and percentages for categorical variables. No statistical inference tests were conducted. Analyses were performed using SAS version 9.4 (SAS Institute, Cary, NC, USA).

## Results

### Patient population

The full cohort included 19,558 patients with a median (Q1; Q3) age of 66 (51; 74) years (Table 1). A slight majority of patients were female (55.4%), White (52.3%), enrolled in Medicare (54.7%), and aged ≥ 65 years (52.0%). When categorized by acute COVID-19 severity, 15,457 patients (79.0%) did not require hospitalization, 2916 patients (14.9%) were hospitalized without ICU admission, and 1185 patients (6.1%) were admitted to the ICU. Male patients, Black patients, and those aged ≥ 65 years were observed in higher proportions among the cohorts that were hospitalized (with or without ICU admission) for COVID-19.

**Table 1:** Baseline demographic characteristics

Characteristic	All Patients (*N* = 19,558)	Age Group, years		Disposition During Acute COVID-19 Illness		
		**18–64 (*n* = 9381)**	** ≥ 65 (*n* = 10,177)**	**No Hospitalization (*n* = 15,457)**	**Hospitalization Without ICU Admission (*n* = 2916)**	**ICU Admission (*n* = 1185)**
Sex, n (%)						
Female	10,844 (55.4)	5186 (55.3)	5658 (55.6)	8755 (56.6)	1522 (52.2)	567 (47.8)
Male	8714 (44.6)	4195 (44.7)	4519 (44.4)	6702 (43.4)	1394 (47.8)	618 (52.2)
Age, y						
Mean ± SD	61.9 ± 16.4	47.9 ± 11.7	74.9 ± 6.6	59.8 ± 16.5	70.5 ± 13.5	69.0 ± 12.0
Median (Q1; Q3)	66 (51; 74)	50 (40; 58)	74 (69; 79)	62 (48; 72)	73 (64; 80)	71 (62; 77)
Age group, y, n (%)						
18–29	858 (4.4)	858 (9.2)	0 (0)	817 (5.3)	33 (1.1)	8 (0.7)
30–49	3596 (18.4)	3596 (38.3)	0 (0)	3327 (21.5)	204 (7.0)	65 (5.5)
50–64	4927 (25.2)	4927 (52.5)	0 (0.0)	4126 (26.7)	522 (17.9)	279 (23.5)
65–74	5640 (28.8)	0 (0)	5640 (55.4)	4293 (27.8)	912 (31.3)	435 (36.7)
≥ 75	4537 (23.2)	0 (0)	4537 (44.6)	2894 (18.7)	1245 (42.7)	398 (33.6)
Race or ethnicity, n (%)						
White	10,232 (52.3)	4679 (49.9)	5553 (54.6)	8410 (54.4)	1366 (46.8)	456 (38.5)
Black	3398 (17.4)	1564 (16.7)	1834 (18.0)	2391 (15.5)	716 (24.6)	291 (24.6)
Hispanic	3884 (19.9)	2096 (22.3)	1788 (17.6)	3050 (19.7)	539 (18.5)	295 (24.9)
Asian	768 (3.9)	357 (3.8)	411 (4.0)	590 (3.8)	121 (4.2)	57 (4.8)
Unknown	1276 (6.5)	685 (7.3)	591 (5.8)	1016 (6.6)	174 (6.0)	86 (7.3)
Geographic division, n (%)						
New England	1729 (8.8)	648 (6.9)	1081 (10.6)	1356 (8.8)	270 (9.3)	103 (8.7)
Mid-Atlantic	5299 (27.1)	2127 (22.7)	3172 (31.2)	4386 (28.4)	738 (25.3)	175 (14.8)
East North Central	2409 (12.3)	1320 (14.1)	1089 (10.7)	1804 (11.7)	430 (14.8)	175 (14.8)
West North Central	988 (5.1)	677 (7.2)	311 (3.1)	790 (5.1)	133 (4.6)	65 (5.5)
South Atlantic	3983 (20.4)	1963 (20.9)	2020 (19.9)	3046 (19.7)	621 (21.3)	316 (26.7)
East South Central	527 (2.7)	301 (3.2)	226 (2.2)	399 (2.6)	92 (3.2)	36 (3.0)
West South Central	1790 (9.2)	1099 (11.7)	691 (6.8)	1375 (8.9)	269 (9.2)	146 (12.3)
Mountain	1576 (8.1)	677 (7.2)	899 (8.8)	1253 (8.1)	225 (7.7)	98 (8.3)
Pacific	1218 (6.2)	556 (5.9)	662 (6.5)	1019 (6.6)	131 (4.5)	68 (5.7)
Insurance, n (%)						
Commercial	8857 (45.3)	8115 (86.5)	742 (7.3)	7991 (51.7)	584 (20.0)	282 (23.8)
Medicare	10,696 (54.7)	1265 (13.5)	9431 (92.7)	7462 (48.3)	2332 (80.0)	902 (76.1)
Commercial/Medicare	3 (0.02)	0 (0.0)	3 (0.03)	2 (0.01)	0 (0.0)	1 (0.1)
Unknown	2 (0.01)	1 (0.01)	1 (0.01)	2 (0.01)	0 (0.0)	0 (0.0)

*ICU* intensive care unit, *Q1* quartile 1, *Q3* quartile 3

The majority of patients (81.5%) had ≥ 2 high-risk conditions, and 7.7% of patients had ≥ 8 such conditions (Table 2). The most common high-risk conditions in the overall population were immunocompromised state (71.2%), hypertension (60.5%), and age ≥ 65 years (52.0%). Most conditions were more common among older patients and among those who were hospitalized (with or without ICU admission) for acute COVID-19.

**Table 2:** Baseline medical characteristics, including presence of high-risk conditions^a^

Characteristic, n (%)	All Patients (*N* = 19,558)	Age Group, years		Disposition During Acute COVID-19 Illness		
		**18–64 (*n* = 9381)**	** ≥ 65 (*n* = 10,177)**	**No Hospitalization (*n* = 15,457)**	**Hospitalization Without ICU Admission (*n* = 2916)**	**ICU Admission (*n* = 1185)**
High-risk condition						
Aged ≥ 65 years	10,177 (52.0)	0 (0.0)	10,177 (100)	7187 (46.5)	2157 (74.0)	833 (70.3)
Cancer history	2933 (15.0)	722 (7.7)	2211 (21.7)	2214 (14.3)	533 (18.3)	186 (15.7)
Chronic kidney disease	2783 (14.2)	612 (6.5)	2171 (21.3)	1646 (10.7)	832 (28.5)	305 (25.7)
Chronic liver disease^b^	218 (1.1)	88 (0.9)	130 (1.3)	139 (0.9)	56 (1.9)	23 (1.9)
Chronic lung disease^c^	2566 (13.1)	915 (9.8)	1651 (16.2)	1755 (11.4)	613 (21.0)	198 (16.7)
Dementia or other neurologic condition	1121 (5.7)	237 (2.5)	884 (8.7)	716 (4.6)	320 (11.0)	85 (7.2)
Diabetes	6081 (31.1)	2060 (22.0)	4021 (39.5)	4168 (27.0)	1319 (45.2)	594 (50.1)
Down syndrome	4 (0.02)	4 (0.04)	0 (0.0)	2 (0.01)	1 (0.03)	1 (0.1)
Heart condition^d^	6347 (32.5)	1593 (17.0)	4754 (46.7)	4375 (28.3)	1472 (50.5)	500 (42.2)
HIV	158 (0.8)	107 (1.1)	51 (0.5)	120 (0.8)	28 (1.0)	10 (0.8)
Hypertension	11,823 (60.5)	3966 (42.3)	7857 (77.2)	8558 (55.4)	2323 (79.7)	942 (79.5)
Immunocompromised state^e^	13,931 (71.2)	6464 (68.9)	7467 (73.4)	10,848 (70.2)	2222 (76.2)	861 (72.7)
Mental health condition^f^	3214 (16.4)	1615 (17.2)	1599 (15.7)	2453 (15.9)	570 (19.6)	191 (16.1)
Overweight or obesity	7361 (37.6)	3742 (39.9)	3619 (35.6)	5615 (36.3)	1209 (41.5)	537 (45.3)
Pregnancy or recent pregnancy^g^	375 (1.9)	375 (4.0)	0 (0.0)	331 (2.1)	41 (1.4)	3 (0.3)
Sickle cell disease or thalassemia	72 (0.4)	45 (0.5)	27 (0.3)	57 (0.4)	12 (0.4)	3 (0.3)
Smoking, current or previous	3463 (17.7)	1274 (13.6)	2189 (21.5)	2393 (15.5)	806 (27.6)	264 (22.3)
Solid organ or blood stem cell transplant^h^	34 (0.2)	22 (0.2)	12 (0.1)	21 (0.1)	12 (0.4)	1 (0.1)
Stroke or cerebrovascular disease	1730 (8.9)	326 (3.5)	1404 (13.8)	1152 (7.5)	440 (15.1)	138 (11.7)
Substance use disorder^i^	589 (3.0)	317 (3.4)	272 (2.7)	413 (2.7)	145 (5.0)	31 (2.6)
Tuberculosis	124 (0.6)	68 (0.7)	56 (0.6)	97 (0.6)	22 (0.8)	5 (0.4)
Number of high-risk conditions, mutually exclusive						
1	3623 (18.5)	3147 (33.6)	476 (4.7)	3322 (21.5)	205 (7.0)	96 (8.1)
2	3229 (16.5)	2376 (25.3)	853 (8.4)	2878 (18.6)	235 (8.1)	116 (9.8)
3	2999 (15.3)	1590 (17.0)	1409 (13.8)	2496 (16.2)	340 (11.7)	163 (13.8)
4	2626 (13.4)	936 (10.0)	1690 (16.6)	2087 (13.5)	383 (13.1)	156 (13.2)
5	2377 (12.2)	573 (6.1)	1804 (17.7)	1729 (11.2)	442 (15.2)	206 (17.4)
6	1880 (9.6)	356 (3.8)	1524 (15.0)	1293 (8.4)	413 (14.2)	174 (14.7)
7	1327 (6.8)	207 (2.2)	1120 (11.0)	835 (5.4)	370 (12.7)	122 (10.3)
≥ 8	1497 (7.7)	196 (2.1)	1301 (12.8)	817 (5.3)	528 (18.1)	152 (12.8)
Care setting of COVID-19 diagnosis						
Inpatient	3385 (17.3)	847 (9.0)	2538 (24.9)	0 (0.0)	2431 (83.4)	954 (80.5)
Outpatient	16,173 (82.7)	8534 (91.0)	7639 (75.1)	15,457 (100)	485 (16.6)	231 (19.5)
Disposition during acute COVID-19 illness						
No hospitalization	15,457 (79.0)	8270 (88.2)	7187 (70.6)			
Hospitalization without ICU admission	2916 (14.9)	759 (8.1)	2157 (21.2)			
ICU admission	1185 (6.1)	352 (3.8)	833 (8.2)			
COVID-19 inpatient stay that overlaps acute and post-acute phases; > 30 days	143 (0.7)	35 (0.4)	108 (1.1)	0 (0.0)	36 (1.2)	107 (9.0)

*ICU* intensive care unit

^a^Conditions placing individuals at high risk of developing severe illness from COVID-19 were determined by the Centers for Disease Control and Prevention [29]

^b^Includes cirrhosis, nonalcoholic fatty liver disease, alcoholic liver disease, and autoimmune hepatitis

^c^Includes moderate to severe asthma, bronchiectasis, bronchopulmonary dysplasia, chronic obstructive pulmonary disease, emphysema, chronic bronchitis, interstitial lung disease, pulmonary fibrosis, cystic fibrosis, pulmonary embolism, and pulmonary hypertension

^d^Includes heart failure, coronary artery disease, and cardiomyopathies

^e^Included both primary immunocompromised state (genetic condition) and secondary or acquired immunocompromised state (prolonged use of medication that weakens the immune system, such as corticosteroids or antimetabolites). Qualifying diagnoses included HIV/AIDS, solid malignancy, bone marrow transplant, organ transplant, rheumatologic/inflammatory conditions, primary immunodeficiency, chronic kidney disease or end-stage renal disease, and hematologic malignancy

^f^Included mood disorder or schizophrenia spectrum disorder

^g^Recent pregnancy defined as a pregnancy occurring within 42 days before the index date; excludes women aged ≥ 45 years

^h^Including bone marrow transplant

^i^Included alcohol, opioid, or cocaine abuse

Most patients (82.7%) were diagnosed with COVID-19 in the outpatient setting (Table 2). Of those who were hospitalized at any time during the acute phase, 143 patients (0.7%) had hospital stays spanning > 30 days after the index date and therefore overlapping the acute and post-acute phases. Few patients (0.5% of the overall cohort) died during the post-acute phase. A full list of reasons for exclusion from the analysis is shown in Table S1.

### Diagnoses

**Fig. 2:**
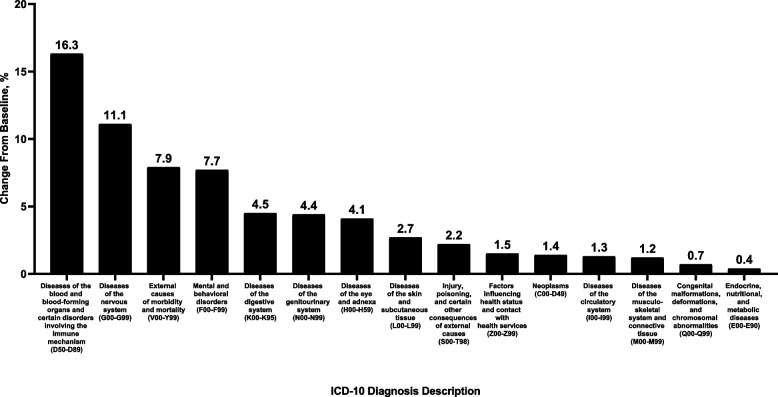
Percentage change from baseline to post-acute phase in ICD-10 diagnoses in the overall population (*N* = 19,558). Diagnosis codes (shown in parentheses) include those with a prevalence of ≥ 2% in the baseline population and with an increase from baseline to post-acute phases. Code ranges, raw values at baseline and post-acute phases, and diagnoses decreasing from baseline to post-acute phases are shown in Table 3. ICD-10, International Classification of Diseases, Tenth Revision

Between the baseline and post-acute phases, the frequency of individual ICD-10-CM diagnosis codes increased within several chapters (Fig. 2). The greatest percentage increase was observed in the ICD-10 chapter of “diseases of the blood and blood-forming organs and certain disorders involving the immune mechanism” (+ 16.3%), followed by “diseases of the nervous system” (+ 11.1%), “external causes of morbidity and mortality” (+ 7.9%), and “mental and behavioral disorders” (+ 7.7%).

Frequencies of other disorders increased more modestly or decreased (Table 3); the greatest percentage decrease was observed in the chapter of “diseases of the respiratory system” (–18.3%). Within this chapter, the decrease was driven specifically by a drop in frequency of the subchapters acute upper and lower respiratory infections (–56.6% and –69.3%, respectively), which outweighed increases in frequency of other respiratory conditions, such as “other respiratory diseases principally affecting the interstitium” (+ 59.8%; Table S2 and Fig. S1).

**Table 3:** Diagnoses during the baseline and post-acute phases in the overall population (*N* = 19,558)^a^

ICD-10 Diagnosis Description	ICD-10 Diagnosis Code	Baseline Phase, n (%)	Post-Acute Phase, n (%)	Change From Baseline to Post-Acute Phase, Δ n (% Change)
Diseases of the blood and blood-forming organs and certain disorders involving the immune mechanism	D50–D89	5010 (25.6)	5825 (29.8)	815 (16.3)
Diseases of the nervous system	G00–G99	7345 (37.6)	8159 (41.7)	814 (11.1)
External causes of morbidity and mortality	V00–Y99	1503 (7.7)	1621 (8.3)	118 (7.9)
Mental and behavioral disorders	F00–F99	6875 (35.2)	7404 (37.9)	529 (7.7)
Diseases of the digestive system	K00–K95	8361 (42.8)	8736 (44.7)	375 (4.5)
Diseases of the genitourinary system	N00–N99	9532 (48.7)	9954 (50.9)	422 (4.4)
Diseases of the eye and adnexa	H00–H59	7332 (37.5)	7630 (39.0)	298 (4.1)
Diseases of the skin and subcutaneous tissue	L00–L99	7205 (36.8)	7396 (37.8)	191 (2.7)
Injury, poisoning, and certain other consequences of external causes	S00–T98	5445 (27.8)	5564 (28.4)	119 (2.2)
Factors influencing health status and contact with health services	Z00–Z99	18,359 (93.9)	18,629 (95.3)	270 (1.5)
Neoplasms	C00–D49	6066 (31.0)	6152 (31.5)	86 (1.4)
Diseases of the circulatory system	I00–I99	13,381 (68.4)	13,551 (69.3)	170 (1.3)
Diseases of the musculoskeletal system and connective tissue	M00–M99	12,618 (64.5)	12,766 (65.3)	148 (1.2)
Congenital malformations, deformations, and chromosomal abnormalities	Q00–Q99	730 (3.7)	735 (3.8)	5 (0.7)
Endocrine, nutritional, and metabolic diseases	E00–E90	15,251 (78.0)	15,305 (78.3)	54 (0.4)
Symptoms, signs, and abnormal clinical and laboratory findings not elsewhere classified	R00–R99	16,977 (86.8)	16,225 (83.0)	–752 (–4.4)
Diseases of the ear and mastoid process	H60–H95	3041 (15.5)	2902 (14.8)	–139 (–4.6)
Certain infectious and parasitic diseases	A00–B99	6543 (33.5)	5701 (29.1)	–842 (–12.9)
Diseases of the respiratory system	J00–J99	11,296 (57.8)	9227 (47.2)	–2069 (–18.3)

*ICD-10* International Classification of Diseases, Tenth Revision

^a^The baseline phase was the 12 months before the index date, and the post-acute phase spanned from 1 to 13 months after the index date

### Medication use

**Fig. 3:**
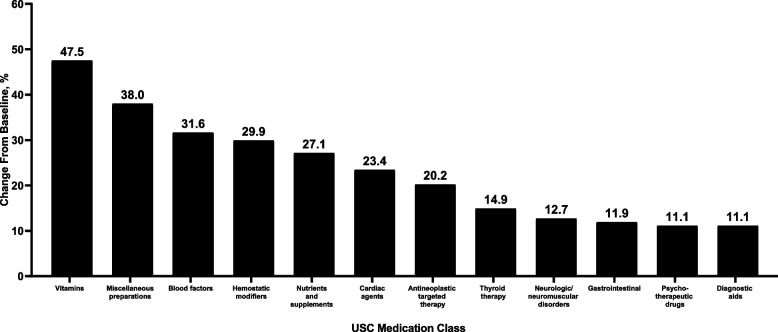
Percentage change from baseline to post-acute phase in prescription frequency in the overall population (*N* = 19,558). Medication classes shown are according to USC designations; included classes are those prescribed to ≥ 2% of the baseline population and with a percentage change from baseline to post-acute phase of ≥ 10%. Percentage changes from baseline to post-acute phase according to age group and disease severity are shown in Table 4, and raw values during baseline and post-acute phases for all medication classes regardless of percentage change are shown for the overall population in Table S3. USC, Uniform System of Classification

Prescription frequencies across several medication classes increased between baseline and the post-acute phase (Fig. 3). The greatest percentage increases were observed for vitamins (+ 47.5%), miscellaneous preparations (+ 38.0%), blood factors (+ 31.6%), and hemostatic modifiers (+ 29.9%). Among medication classes with increases of ≥ 10% in the overall population, increases in prescription frequency were observed across all ages and severities (Tables 4 and 5).

**Table 4:** Medications with frequency Increases ≥ 10% from the baseline to the post-acute phase^a^ by age group

USC Medication Class Description	All Patients (*N* = 19,558)			Patients Aged 18–64 Years (*n* = 9381)			Patients Aged ≥ 65 Years (*n* = 10,177)		
	**Baseline Phase, n (%)**	**Post-Acute Phase, n (%)**	**Change From Baseline to Post-Acute Phase, Δ (% Change)**	**Baseline Phase, n (%)**	**Post-Acute Phase, n (%)**	**Change From Baseline to Post-Acute Phase, Δ (% Change)**	**Baseline Phase, n (%)**	**Post-Acute Phase, n (%)**	**Change From Baseline to Post-Acute Phase, Δ (% Change)**
Vitamins	771 (3.9)	1137 (5.8)	366 (47.5)	576 (6.1)	663 (7.1)	87 (15.1)	195 (1.9)	474 (4.7)	279 (143.1)
Miscellaneous preparations	831 (4.2)	1147 (5.9)	316 (38.0)	407 (4.3)	546 (5.8)	139 (34.2)	424 (4.2)	601 (5.9)	177 (41.7)
Blood factors	675 (3.5)	888 (4.5)	213 (31.6)	313 (3.3)	368 (3.9)	55 (17.6)	362 (3.6)	520 (5.1)	158 (43.6)
Hemostatic modifiers	2093 (10.7)	2719 (13.9)	626 (29.9)	563 (6.0)	759 (8.1)	196 (34.8)	1530 (15.0)	1960 (19.3)	430 (28.1)
Nutrients and supplements	980 (5.0)	1246 (6.4)	266 (27.1)	340 (3.6)	405 (4.3)	65 (19.1)	640 (6.3)	841 (8.3)	201 (31.4)
Cardiac agents	1203 (6.2)	1485 (7.6)	282 (23.4)	459 (4.9)	529 (5.6)	70 (15.3)	744 (7.3)	956 (9.4)	212 (28.5)
Antineoplastic targeted therapy	536 (2.7)	644 (3.3)	108 (20.1)	183 (2.0)	227 (2.4)	44 (24.0)	353 (3.5)	417 (4.1)	64 (18.1)
Thyroid therapy	2222 (11.4)	2553 (13.1)	331 (14.9)	797 (8.5)	916 (9.8)	119 (14.9)	1425 (14.0)	1637 (16.1)	212 (14.9)
Neurologic/neuromuscular disorders	3790 (19.4)	4271 (21.8)	481 (12.7)	1682 (17.9)	1851 (19.7)	169 (10.0)	2108 (20.7)	2420 (23.8)	312 (14.8)
Gastrointestinal	5002 (25.6)	5595 (28.6)	593 (11.9)	2067 (22.0)	2234 (23.8)	167 (8.1)	2935 (28.8)	3361 (33.0)	426 (14.5)
Psychotherapeutic drugs	5876 (30.0)	6530 (33.4)	654 (11.1)	3150 (33.6)	3366 (35.9)	216 (6.9)	2726 (26.8)	3164 (31.1)	438 (16.1)
Diagnostic aids	3288 (16.8)	3652 (18.7)	364 (11.1)	1428 (15.2)	1582 (16.9)	154 (10.8)	1860 (18.3)	2070 (20.3)	210 (11.3)

*USC* Uniform System of Classification

^a^The baseline period was the 12 months before the index date, and the post-acute phase spanned from 1 to 13 months after the index date

**Table 5:** Medications with frequency increases ≥ 10% from baseline to post-acute phase^a^ by disposition during acute COVID-19

USC Medication Class Description	No Hospitalization (*n* = 15,457)			Hospitalization Without ICU Admission (*n* = 2916)			ICU Admission (*n* = 1185)		
	**Baseline Phase, n (%)**	**Post-Acute Phase, n (%)**	**Change From Baseline to Post-Acute Phase, Δ (% Change)**	**Baseline Phase, n (%)**	**Post-Acute Phase, n (%)**	**Change From Baseline to Post-Acute Phase, Δ (% Change)**	**Baseline Phase, n (%)**	**Post-Acute Phase, n (%)**	**Change From Baseline to Post-Acute Phase, Δ (% Change)**
Vitamins	619 (4.0)	891 (5.8)	272 (43.9)	100 (3.4)	166 (5.7)	66 (66.0)	52 (4.4)	80 (6.8)	28 (53.8)
Miscellaneous preparations	603 (3.9)	792 (5.1)	189 (31.3)	161 (5.5)	250 (8.6)	89 (55.3)	67 (5.7)	105 (8.9)	38 (56.7)
Blood factors	499 (3.2)	627 (4.1)	128 (25.7)	134 (4.6)	185 (6.3)	51 (38.1)	42 (3.5)	76 (6.4)	34 (81.0)
Hemostatic modifiers	1366 (8.8)	1618 (10.5)	252 (18.4)	533 (18.3)	765 (26.2)	232 (43.5)	194 (16.4)	336 (28.4)	142 (73.2)
Nutrients and supplements	625 (4.0)	771 (5.0)	146 (23.4)	258 (8.8)	325 (11.1)	67 (26.0)	97 (8.2)	150 (12.7)	53 (54.6)
Cardiac agents	808 (5.2)	968 (6.3)	160 (19.8)	279 (9.6)	352 (12.1)	73 (26.2)	116 (9.8)	165 (13.9)	49 (42.2)
Antineoplastic targeted therapy	384 (2.5)	454 (2.9)	70 (18.2)	109 (3.7)	135 (4.6)	26 (23.9)	43 (3.6)	55 (4.6)	12 (27.9)
Thyroid therapy	1722 (11.1)	1958 (12.7)	236 (13.7)	361 (12.4)	427 (14.6)	66 (18.3)	139 (11.7)	168 (14.2)	29 (20.9)
Neurologic/neuromuscular disorders	2736 (17.7)	3065 (19.8)	329 (12.0)	749 (25.7)	836 (28.7)	87 (11.6)	305 (25.7)	370 (31.2)	65 (21.3)
Gastrointestinal	3769 (24.4)	4097 (26.5)	328 (8.7)	879 (30.1)	1015 (34.8)	136 (15.5)	354 (29.9)	483 (40.8)	129 (36.4)
Psychotherapeutic drugs	4697 (30.4)	5077 (32.8)	380 (8.1)	871 (29.9)	1043 (35.8)	172 (19.7)	308 (26.0)	410 (34.6)	102 (33.1)
Diagnostic aids	2394 (15.5)	2614 (16.9)	220 (9.2)	611 (21.0)	710 (24.3)	99 (16.2)	283 (23.9)	328 (27.7)	45 (15.9)

*ICU* intensive care unit, USC Uniform System of Classification

^a^The baseline period was the 12 months before the index date, and the post-acute phase spanned from 1 to 13 months after the index date

Other prescription frequencies increased marginally or decreased (Table S3). The greatest overall decreases were within the classes of cough/cold/flu preparations (− 73.7%), antimalarials (–54.5%), and antivirals (–40.4%).

### Medical care and hospitalizations

Increases in total and per-patient inpatient and outpatient visits and procedures were observed during the post-acute phase compared with baseline (Tables 6 and 7). The greatest percentage increases were observed for LOS (including total days [+ 165.6%] and mean days per patient [+ 132.9%]), inpatient lab tests (+ 105.1%), and all-cause readmissions within 30 days (+ 249.9%). For these 3 variables, increases from baseline to the post-acute phase were greatest among patients who were admitted to the ICU during acute COVID-19, but were substantial even among those who did not require hospitalization for acute COVID-19. The number of patients with ICU visits was relatively consistent from baseline to the post-acute phase in the overall population but increased by 43.2% among those who were admitted to the ICU during acute COVID-19 illness. Among all age and severity categories, the numbers of patients visiting the ED decreased. Regarding hospital discharge status, percentages of all discharges to another hospital department or facility (such as hospice care or a skilled nursing facility) were low during the baseline phase owing to exclusion criteria but substantially increased among both age groups (up to a 13,000% increase in intra-institution transfers among patients aged ≥ 65 years; Table S4).

**Table 6:** Healthcare resource use during baseline and post-acute phases^a^ in the overall population and by age

Visit or Procedure	All Patients (*N* = 19,558)			Patients Aged 18–64 Years (*n* = 9381)			Patients Aged ≥ 65 Years (*n* = 10,177)		
	**Baseline Phase**	**Post-Acute Phase**	**Change From Baseline to Post-Acute Phase, Δ (% Change)**	**Baseline Phase**	**Post-Acute Phase**	**Change From Baseline to Post-Acute Phase, Δ (% Change)**	**Baseline Phase**	**Post-Acute Phase**	**Change From Baseline to Post-Acute Phase, Δ (% Change)**
Outpatient lab tests									
Tests, n	36,229	47,716	11,487 (31.7)	16,008	21,590	5582 (34.9)	20,221	26,126	5905 (29.2)
Patients, n	12,453	13,565	1112 (8.9)	5809	6279	470 (8.1)	6644	7286	642 (9.7)
Mean ± SD	1.9 ± 2.6	2.4 ± 3.3	0.6	1.7 ± 2.5	2.3 ± 3.2	0.6	2.0 ± 2.7	2.6 ± 3.3	0.6
Median (Q1; Q3)	1 (0; 3)	1 (0; 3)	0	1 (0; 2)	1 (0; 3)	0	1 (0; 3)	2 (0; 4)	1
Outpatient visits (specialist or nonspecialist)									
Visits, n	488,305	613,201	124,896 (25.6)	190,988	235,557	44,569 (23.3)	297,317	377,644	80,327 (27.0)
Patients, n	19,413	19,223	–190 (–1.0)	9357	9173	–184 (–2.0)	10,056	10,050	–6 (–0.1)
Mean ± SD	25.0 ± 29.0	31.4 ± 36.3	6.4	20.4 ± 25.5	25.1 ± 30.5	4.8	29.2 ± 31.3	37.1 ± 40.1	7.9
Median (Q1; Q3)	17 (9; 31)	20 (10; 39)	3	13 (7; 24)	16 (8; 31)	3	20 (11; 36)	26 (13; 47)	6
Emergency department visits									
Visits, n	11,168	9205	–1963 (–17.6)	5514	4418	–1096 (–19.9)	5654	4787	–867 (–15.3)
Patients, n	5943	4756	–1187 (–20.0)	2830	2149	–681 (–24.1)	3113	2607	–506 (–16.3)
Mean ± SD	0.6 ± 1.6	0.5 ± 1.9	–0.1	0.6 ± 1.9	0.5 ± 2.3	–0.1	0.6 ± 1.2	0.5 ± 1.3	–0.1
Median (Q1; Q3)	0 (0; 1)	0 (0; 0)	0	0 (0; 1)	0 (0; 0)	0	0 (0; 1)	0 (0; 1)	0
Prescription classes									
Prescriptions, n	110,001	111,618	1617 (1.5)	49,424	48,329	–1095 (–2.2)	60,577	63,289	2712 (4.5)
Patients, n	17,609	17,520	–89 (–0.5)	8795	8550	–245 (–2.8)	8814	8970	156 (1.8)
Mean ± SD	5.6 ± 4.0	5.7 ± 4.1	0.1	5.3 ± 3.8	5.2 ± 4.0	–0.1	6.0 ± 4.1	6.2 ± 4.2	0.3
Median (Q1; Q3)	5 (3; 8)	5 (3; 8)	0	5 (2; 7)	4 (2; 7)	–1	6 (3; 9)	6 (3; 9)	0
Inpatient visits									
Visits, n	3642	5301	1659 (45.6)	1314	1528	214 (16.3)	2328	3773	1445 (62.1)
Patients, n	2523	2877	354 (14.0)	884	878	–6 (–0.7)	1639	1999	360 (22.0)
Mean ± SD	0.2 ± 0.6	0.3 ± 0.9	0.1	0.1 ± 0.6	0.2 ± 0.8	0.0	0.2 ± 0.6	0.4 ± 1.0	0.1
Median (Q1; Q3)	0 (0; 0)	0 (0; 0)	0	0 (0; 0)	0 (0; 0)	0	0 (0; 0)	0 (0; 0)	0
Inpatient lab tests									
Tests, n	851	1745	894 (105.1)	299	397	98 (32.8)	552	1348	796 (144.2)
Patients, n	379	521	142 (37.5)	108	149	41 (38.0)	271	372	101 (37.3)
Mean ± SD	0.0 ± 0.5	0.1 ± 1.0	0.1	0.0 ± 0.6	0.0 ± 0.6	0.0	0.1 ± 0.5	0.1 ± 1.3	0.1
Median (Q1; Q3)	0 (0; 0)	0 (0; 0)	0	0 (0; 0)	0 (0; 0)	0	0 (0; 0)	0 (0; 0)	0
Length of hospital stay									
Days, n	18,941	50,310	31,369 (165.6)	7313	12,290	4977 (68.1)	11,628	38,020	26,392 (227.0)
Patients, n	2523	2877	354 (14.0)	884	878	–6 (–0.7)	1639	1999	360 (22.0)
Mean ± SD	7.5 ± 10.1	17.5 ± 25.2	10.0 (132.9)	8.3 ± 12.7	14.0 ± 25.9	5.7 (69.2)	7.1 ± 8.3	19.0 ± 24.8	11.9 (168.1)
Median (Q1; Q3)	4 (3; 8)	8 (4; 21)	4	4 (3; 8)	5 (3; 13)	1	5 (3; 8)	9 (4; 24)	4
Length of ICU stay									
Days, n	937	1068	131 (14.0)	298	396	98 (32.9)	639	672	33 (5.2)
Patients, n	742	743	1 (0.1)	214	217	3 (1.4)	528	526	–2 (–0.4)
Mean ± SD	0.4 ± 0.7	0.4 ± 0.9	0.0 (0.0)	0.3 ± 0.8	0.5 ± 1.3	0.1 (33.8)	0.4 ± 0.7	0.3 ± 0.7	–0.1 (–13.8)
Median (Q1; Q3)	0 (0; 1)	0 (0; 1)	0	0 (0; 0)	0 (0; 0)	0	0 (0; 1)	0 (0; 1)	0
Patients with invasive mechanical ventilation use, n (%)	165 (0.8)	210 (1.1)	45 (27.3)	58 (0.6)	75 (0.8)	17 (29.3)	107 (1.1)	135 (1.3)	28 (26.2)
Patients with noninvasive mechanical ventilation use, n (%)	147 (0.8)	173 (0.9)	26 (17.7)	51 (0.5)	60 (0.6)	9 (17.7)	96 (0.9)	113 (1.1)	17 (17.7)
Patients with supplemental oxygen use, n (%)	199 (1.0)	358 (1.8)	159 (79.9)	53 (0.6)	90 (1.0)	37 (69.8)	146 (1.4)	268 (2.6)	122 (83.6)
Patients with readmission within 30 days, n (%)	327 (1.7)	1144 (5.9)	817 (249.9)	131 (1.4)	220 (2.3)	89 (67.9)	196 (1.9)	924 (9.1)	728 (371.4)

For visits, tests, prescriptions, and procedures, means or percentages were calculated using the total number of patients within the cohort as the denominator. For length of hospital stay and ICU stay, means were calculated as the total number of days divided by the number of patients with any inpatient hospital stay

*ICU* intensive care unit, *Q1* quartile 1, *Q3* quartile 3

^a^The baseline period was the 12 months before the index date, and the post-acute phase spanned from 1 to 13 months after the index date

**Table 7:** Healthcare resource use during baseline and post-acute phases^a^ stratified by disposition during acute COVID-19

Visit or Procedure	No Hospitalization (*n* = 15,457)			Hospitalization Without ICU Admission (*n* = 2916)			ICU Admission (*n* = 1185)		
	**Baseline Phase**	**Post-Acute Phase**	**Change From Baseline to Post-Acute Phase, Δ (% Change)**	**Baseline Phase**	**Post-Acute Phase**	**Change From Baseline to Post-Acute Phase, Δ (% Change)**	**Baseline Phase**	**Post-Acute Phase**	**Change From Baseline to Post-Acute Phase, Δ (% Change)**
Outpatient lab tests									
Tests, n	28,074	37,092	9018 (32.1)	5694	7323	1629 (28.6)	2461	3301	840 (34.1)
Patients, n	9941	10,762	821 (8.3)	1778	1975	197 (11.1)	734	828	94 (12.8)
Mean ± SD	1.8 ± 2.5	2.4 ± 3.2	0.6	2.0 ± 3.0	2.5 ± 3.6	0.6	2.1 ± 3.3	2.8 ± 3.6	0.7
Median (Q1; Q3)	1 (0; 3)	1 (0; 3)	0	1 (0; 3)	1 (0; 3.5)	0	1 (0; 3)	2 (0; 4)	1
Outpatient visits (specialist or nonspecialist)									
Visits, n	357,264	424,453	67,189 (18.8)	95,986	132,282	36,296 (37.8)	35,055	56,466	21,411 (61.1)
Patients, n	15,362	15,183	–179 (–1.2)	2882	2878	–4 (–0.1)	1169	1162	–7 (–0.6)
Mean ± SD	23.1 ± 25.3	27.5 ± 30.5	4.3	32.9 ± 39.0	45.4 ± 49.8	12.4	29.6 ± 40.2	47.7 ± 50.6	18.1
Median (Q1; Q3)	16 (8; 29)	18 (9; 35)	2	21 (11; 41)	30 (14; 57)	9	17 (9; 34)	33 (17; 61)	16
Emergency department visits									
Visits, n	7753	6196	–1557 (–20.1)	2615	2191	–424 (–16.2)	800	818	18 (2.3)
Patients, n	4368	3427	–941 (–21.5)	1168	968	–200 (–17.1)	407	361	–46 (–11.3)
Mean ± SD	0.5 ± 1.4	0.4 ± 1.5	–0.1	0.9 ± 2.3	0.8 ± 2.0	–0.1	0.7 ± 1.5	0.7 ± 4.2	< 0.1
Median (Q1; Q3)	0 (0; 1)	0 (0; 0)	0	0 (0; 1)	0 (0; 1)	0	0 (0; 1)	0 (0; 1)	0
Prescription classes									
Prescriptions, n	83,348	82,917	–431 (–0.5)	18,783	20,082	1299 (6.9)	7870	8619	749 (9.5)
Patients, n	13,958	13,821	–137 (–1.0)	2564	2608	44 (1.7)	1087	1091	4 (0.4)
Mean ± SD	5.4 ± 3.8	5.4 ± 3.9	–0.0	6.4 ± 4.4	6.9 ± 4.5	0.4	6.6 ± 4.3	7.3 ± 4.5	0.6
Median (Q1; Q3)	5 (3; 8)	5 (2; 8)	0	6 (3; 9)	7 (4; 10)	1	6 (4; 9)	7 (4; 10)	1
Inpatient visits									
Visits, n	1998	2372	374 (18.7)	1334	1940	606 (45.4)	310	989	679 (219.0)
Patients, n	1528	1526	–2 (–0.1)	808	886	78 (9.7)	187	465	278 (148.7)
Mean ± SD	0.1 ± 0.5	0.2 ± 0.6	0.0	0.5 ± 1.0	0.7 ± 1.5	0.2	0.3 ± 0.8	0.8 ± 1.5	0.6
Median (Q1; Q3)	0 (0; 0)	0 (0; 0)	0	0 (0; 1)	0 (0; 1)	0	0 (0; 0)	0 (0; 1)	0
Inpatient lab tests									
Tests, n	466	684	218 (46.8)	332	504	172 (51.8)	53	557	504 (950.9)
Patients, n	221	240	19 (8.6)	124	170	46 (37.1)	34	111	77 (226.5)
Mean ± SD	0.0 ± 0.3	0.0 ± 0.6	0.0	0.1 ± 1.0	0.2 ± 1.2	0.1	0.0 ± 0.3	0.5 ± 3.1	0.4
Median (Q1; Q3)	0 (0; 0)	0 (0; 0)	0	0 (0; 0)	0 (0; 0)	0	0 (0; 0)	0 (0; 0)	0
Length of hospital stay									
Days, n	10,536	17,390	6854 (65.1)	6639	19,480	12,841 (193.4)	1766	13,440	11,674 (661.0)
Patients, n	1528	1526	–2 (–0.1)	808	886	78 (9.7)	187	465	278 (148.7)
Mean ± SD	6.9 ± 8.7	11.4 ± 16.0	4.5 (65.3)	8.2 ± 12.2	22.0 ± 28.2	13.8 (167.6)	9.4 ± 10.4	28.9 ± 36.0	19.5 (206.1)
Median (Q1; Q3)	4 (3; 7)	5 (3; 12)	1	5 (3; 9)	11 (5; 26)	6	5 (3; 11)	17 (6; 38)	12
Length of ICU stay									
Days, n	470	505	35 (7.4)	365	354	–11 (–3.0)	102	209	107 (104.9)
Patients, n	377	384	7 (1.9)	284	243	–41 (–14.4)	81	116	35 (43.2)
Mean ± SD	0.3 ± 0.7	0.3 ± 0.7	0.02 (7.6)	0.5 ± 0.8	0.4 ± 0.9	–0.1 (–11.6)	0.5 ± 0.8	0.4 ± 1.4	–0.1 (–17.6)
Median (Q1; Q3)	0 (0; 0)	0 (0; 1)	0	0 (0; 1)	0 (0; 1)	0	0 (0; 1)	0 (0; 0)	0
Patients with invasive mechanical ventilation use, n (%)	62 (0.4)	78 (0.5)	16 (25.8)	87 (3.0)	64 (2.2)	–23 (–26.4)	16 (1.4)	68 (5.7)	52 (325.0)
Patients with noninvasive mechanical ventilation use, n (%)	61 (0.4)	61 (0.4)	0 (0.0)	72 (2.5)	50 (1.7)	–22 (–30.6)	14 (1.2)	62 (5.2)	48 (342.9)
Patients with supplemental oxygen use, n (%)	91 (0.6)	107 (0.7)	16 (17.6)	84 (2.9)	148 (5.1)	64 (76.2)	24 (2.0)	103 (8.7)	79 (329.2)
Patients with readmission within 30 days, n (%)	165 (1.1)	348 (2.3)	183 (110.9)	130 (4.5)	477 (16.4)	347 (266.9)	32 (2.7)	319 (26.9)	287 (896.9)

For visits, tests, and prescriptions, means were calculated as the total value divided by the total number of patients within the cohort. For length of hospital stay and ICU stay, means were calculated as the total number of days divided by the number of patients in the cohort with any inpatient hospital stay

*ICU* intensive care unit, *Q1* quartile 1, *Q3* quartile 3

^a^The baseline period was the 12 months before the index date, and the post-acute phase spanned from 1 to 13 months after the index date

### Healthcare costs

In the overall population, total medical costs (including prescription, inpatient, and outpatient costs) increased by 23.0% from the baseline to the post-acute phase (Table 8). Increased percentages were observed across all categories of age and acute COVID-19 severity but were greater among patients aged ≥ 65 years (+ 27.2%) versus younger patients (+ 16.7%) and were greater among patients admitted to the ICU during the acute phase of COVID-19 (+ 70.6%) versus those who were not hospitalized (+ 14.3%) or who were hospitalized without ICU admission (+ 27.9%; Tables 8 and 9).

**Table 8:** Medical costs during baseline and post-acute phases^a^ in the overall population and by age^b^

Cost Description	All Patients (*N* = 19,558)			Patients Aged 18–64 Years (*n* = 9381)			Patients Aged ≥ 65 Years (*n* = 10,177)		
	**Baseline Phase**	**Post-Acute Phase**	**Change From Baseline to Post-Acute Phase, Δ (% Change)**	**Baseline Phase**	**Post-Acute Phase**	**Change From Baseline to Post-Acute Phase, Δ (% Change)**	**Baseline Phase**	**Post-Acute Phase**	**Change From Baseline to Post-Acute Phase, Δ (% Change)**
Inpatient visits									
Total cost	100,310,406	148,478,802	48,168,396 (48.0)	33,383,596	46,783,646	13,400,051 (40.1)	66,926,810	101,695,155	34,768,345 (52.0)
Standard costs, patient n	2523	2877		884	878		1639	1999	
Mean ± SD	39,758 ± 53,027	51,609 ± 67,633	11,851 (29.8)	37,764 ± 45,741	53,284 ± 75,612	15,520 (41.1)	40,834 ± 56,553	50,873 ± 63,821	10,039 (24.6)
Median (Q1; Q3)	23,539 (14,930; 42,393)	29,812 (14,174; 63,142)	6273 (26.6)	21,110 (13,853; 39,804)	28,505 (13,705; 62,482)	7395 (35.0)	24,902 (15,564; 44,262)	30,535 (14,450; 63,458)	5634 (22.6)
Nonzero costs, patient n	2519	2857	338 (13.4)	882	874	–8 (–0.9)	1637	1983	346 (21.1)
Mean ± SD	39,822 ± 53,046	51,970 ± 67,731	12,149 (30.5)	37,850 ± 45,757	53,528 ± 75,699	15,678 (41.4)	40,884 ± 56,570	51,283 ± 63,914	10,400 (25.4)
Median (Q1; Q3)	23,619 (14,979; 42,401)	30,001 (14,418; 63,458)	6381 (27.0)	21,165 (13,872; 39,837)	28,663 (13,872; 62,649)	7498 (35.4)	24,951 (15,604; 44,262)	30,996 (14,591; 63,718)	6045 (24.2)
Readmission									
Total cost	15,508,222	56,595,103	41,086,881 (264.9)	5,968,848	15,288,400	9,319,552 (156.1)	9,539,374	41,306,702	31,767,328 (333.0)
Standard costs, patient n	327	1145		131	220		196	925	
Mean ± SD	47,426 ± 55,733	49,428 ± 72,129	2002 (4.2)	45,564 ± 43,757	69,493 ± 99,048	23,929 (52.5)	48,670 ± 62,550	44,656 ± 63,226	–4014 (–8.3)
Median (Q1; Q3)	28,400 (16,155; 57,798)	22,978 (8525; 60,113)	–5422 (–19.1)	29,053 (16,565; 64,883)	37,735 (13,202; 84,318)	8681 (29.9)	28,348 (16,134; 56,708)	21,351 (7368; 56,103)	–6998 (–24.7)
Nonzero costs, patient n	327	1124	797 (243.7)	131	218	87 (66.4)	196	906	710 (362.2)
Mean ± SD	47,426 ± 55,733	50,352 ± 72,480	2926 (6.2)	45,564 ± 43,757	70,130 ± 99,277	24,567 (53.9)	48,670 ± 62,550	45,592 ± 63,551	–3078 (–6.3)
Median (Q1; Q3)	28,400 (16,155; 57,798)	23,734 (10,067; 62,107)	–4665 (–16.4)	29,053 (16,565; 64,883)	37,895 (13,937; 85,396)	8842 (30.4)	28,348 (16,134; 56,708)	22,147 (8769; 57,039)	–6201 (–21.9)
Outpatient visits									
Total cost	252,738,072	293,241,964	40,503,891 (16.0)	101,032,115	111,061,227	10,029,113 (9.9)	151,705,958	182,180,736	30,474,779 (20.1)
Standard costs, patient n	19,413	19,223		9357	9173		10,056	10,050	
Mean ± SD	13,019 ± 50,071	15,255 ± 50,870	2236 (17.2)	10,797 ± 44,453	12,107 ± 42,523	1310 (12.1)	15,086 ± 54,704	18,127 ± 57,291	3041 (20.2)
Median (Q1; Q3)	4516 (1746; 11,434)	5284 (1981; 13,581)	768 (17.0)	3363 (1302; 8746)	3781 (1398; 9813)	418 (12.4)	5899 (2369; 13,841)	7071 (2792; 16,649)	1172 (19.9)
Nonzero costs, patient n	19,412	19,222	–190 (–1.0)	9357	9173	–184 (–2.0)	10,055	10,049	–6 (–0.1)
Mean ± SD	13,020 ± 50,072	15,256 ± 50,871	2236 (17.2)	10,797 ± 44,453	12,107 ± 42,523	1310 (12.1)	15,088 ± 54,707	18,129 ± 57,293	3042 (20.2)
Median (Q1; Q3)	4516 (1746; 11,435)	5284 (1981; 13,581)	768 (17.0)	3363 (1302; 8746)	3781 (1398; 9813)	418 (12.4)	5901 (2369; 13,844)	7071 (2792; 16,649)	1170 (19.8)
Emergency department visits									
Total cost	20,718,445	16,776,569	–3,941,876 (–19.0)	10,216,455	8,135,400	–2,081,054 (–20.4)	10,501,990	8,641,168	–1,860,822 (–17.7)
Standard costs, patient n	5943	4756		2830	2149		3113	2607	
Mean ± SD	3486 ± 5082	3527 ± 6118	41 (1.2)	3610 ± 6055	3786 ± 7975	176 (4.9)	3374 ± 3993	3315 ± 3972	–59 (–1.8)
Median (Q1; Q3)	2322 (1358; 3981)	2344 (1328; 3971)	22 (0.9)	2265 (1268; 3930)	2385 (1293; 4001)	121 (5.3)	2344 (1484; 4006)	2319 (1360; 3918)	–25 (–1.1)
Nonzero costs, patient n	5935	4752	–1183 (–19.9)	2828	2149	–679 (–24.0)	3107	2603	–504 (–16.2)
Mean ± SD	3491 ± 5083	3530 ± 6120	40 (1.1)	3613 ± 6057	3786 ± 7975	173 (4.8)	3380 ± 3994	3320 ± 3973	–60 (–1.8)
Median (Q1; Q3)	2322 (1362; 3983)	2346 (1332; 3973)	24 (1.0)	2267 (1270; 3932)	2385 (1293; 4001)	118 (5.2)	2352 (1486; 4009)	2322 (1368; 3919)	–30 (–1.3)
Prescription claims									
Total cost	73,381,260	82,891,287	9,510,027 (13.0)	34,332,199	39,019,983	4,687,784 (13.7)	39,049,062	43,871,304	4,822,243 (12.4)
Standard costs, patient n	17,845	17,824		8832	8604		9013	9220	
Mean ± SD	4112 ± 15,111	4651 ± 16,138	538 (13.1)	3887 ± 14,812	4535 ± 16,441	648 (16.7)	4333 ± 15,396	4758 ± 15,851	426 (9.8)
Median (Q1; Q3)	652 (183; 2888)	722 (198; 3598)	70 (10.8)	399 (108; 2052)	458 (116; 2631)	59 (14.7)	942 (316; 3624)	1004 (327; 4345)	62 (6.5)
Nonzero costs, patient n	17,845	17,824	–21 (–0.1)	8832	8604	–228 (–2.6)	9013	9220	207 (2.3)
Mean ± SD	4112 ± 15,111	4651 ± 16,138	538 (13.1)	3887 ± 14,812	4535 ± 16,441	648 (16.7)	4333 ± 15,396	4758 ± 15,851	426 (9.8)
Median (Q1; Q3)	652 (183; 2888)	722 (198; 3598)	70 (10.8)	399 (108; 2052)	458 (116; 2631)	59 (14.7)	942 (316; 3624)	1004 (327; 4345)	62 (6.5)
All medical costs (outpatient, inpatient, and prescription claims)									
Total cost	426,429,738	524,612,052	98,182,314 (23.0)	168,747,909	196,864,856	28,116,948 (16.7)	257,681,830	327,747,196	70,065,366 (27.2)
Standard costs, patient n	19,492	19,375		9381	9264		10,111	10,111	
Mean ± SD	21,877 ± 61,388	27,077 ± 68,249	5200 (23.8)	17,988 ± 54,361	21,251 ± 59,851	3262 (18.1)	25,485 ± 67,055	32,415 ± 74,730	6930 (27.2)
Median (Q1; Q3)	7025 (2579; 19,791)	8045 (2842; 23,394)	1020 (14.5)	5093 (1898; 15,184)	5597 (1934; 17,259)	504 (9.9)	9137 (3605; 24,705)	10,803 (4238; 29,450)	1666 (18.2)
Nonzero costs, patient n	19,492	19,373	–119 (–0.6)	9381	9264	–117 (–1.3)	10,111	10,109	–2 (–0.02)
Mean ± SD	21,877 ± 61,388	27,077 ± 68,249	5200 (23.8)	17,988 ± 54,361	21,251 ± 59,851	3262 (18.1)	25,485 ± 67,055	32,415 ± 74,730	6930 (27.2)
Median (Q1; Q3)	7025 (2579; 19,791)	8045 (2842; 23,394)	1020 (14.5)	5093 (1898; 15,184)	5597 (1934; 17,259)	504 (9.9)	9137 (3605; 24,705)	10,803 (4238; 29,450)	1666 (18.2)

Standard cost patient n’s (used to calculate standard mean and median) reflect the number of patients who had any healthcare encounter for the specified outcome (eg, all patients with ≥ 1 outpatient visit during the specified time frame). Nonzero cost patient n’s (used to calculate nonzero mean and median) reflect the number of patients who had any costs associated with the specified outcome (eg, all patients with costs > 0 attributable to outpatient visits)

^a^The baseline phase was the 12 months before the index date, and the post-acute phase spanned from 1 to 13 months after the index date

^b^All costs are in US dollars rounded to the nearest dollar

**Table 9:** Medical costs during baseline and post-acute phases^a^ stratified by disposition during acute COVID-19^b^

Cost Description	No Hospitalization (*n* = 15,457)			Hospitalization Without ICU Admission (*n* = 2916)			ICU Admission (*n* = 1185)		
	**Baseline Phase**	**Post-Acute Phase**	**Change From Baseline to Post-Acute Phase, Δ (% Change)**	**Baseline Phase**	**Post-Acute Phase**	**Change From Baseline to Post-Acute Phase, Δ (% Change)**	**Baseline Phase**	**Post-Acute Phase**	**Change From Baseline to Post-Acute Phase, Δ (% Change)**
**Inpatient visits**									
Total cost	55,006,160	71,173,890	16,167,729 (29.4)	37,551,499	48,817,949	11,266,450 (30.0)	7,752,746	28,486,963	20,734,217 (267.4)
Standard costs, patient n	1528	1526		808	886		187	465	
Mean ± SD	35,999 ± 45,790	46,641 ± 57,520	10,642 (29.6)	46,475 ± 66,096	55,099 ± 71,721	8625 (18.6)	41,459 ± 40,551	61,262 ± 86,428	19,804 (47.8)
Median (Q1; Q3)	21,462 (14,504; 38,044)	29,627 (14,953; 54,520)	8164 (38.0)	27,718 (16,260; 52,083)	31,155 (13,937; 70,003)	3437 (12.4)	27,769 (15,380; 52,190)	28,301 (6212; 78,651)	532 (1.9)
Nonzero costs, patient n	1527	1520	–7 (–0.5)	805	877	72 (8.9)	187	460	273 (146.0)
Mean ± SD	36,022 ± 45,795	46,825 ± 57,559	10,803 (30.0)	46,648 ± 66,158	55,665 ± 71,869	9017 (19.3)	41,459 ± 40,551	61,928 ± 86,659	20,470 (49.4)
Median (Q1; Q3)	21,480 (14,508; 38,079)	29,780 (15,003; 54,950)	8300 (38.6)	27,784 (16,312; 52,229)	31,884 (14,314; 70,217)	4100 (14.8)	27,769 (15,380; 52,190)	29,569 (7503; 79,361)	1800 (6.5)
**Readmission**									
Total cost	7,197,035	16,934,054	9,737,019 (135.3)	6,826,276	19,945,967	13,119,691 (192.2)	1,484,911	19,715,081	18,230,170 (1227.7)
Standard costs, patient n	165	348		130	477		32	320	
Mean ± SD	43,618 ± 44,151	48,661 ± 63,658	5043 (11.6)	52,510 ± 70,981	41,815 ± 65,602	–10,694 (–20.4)	46,403 ± 34,640	61,610 ± 87,131	15,206 (32.8)
Median (Q1; Q3)	26,212 (14,990; 60,310)	24,379 (15,647; 56,754)	–1834 (–7.0)	29,916 (17,377; 53,143)	19,431 (4351; 51,298)	–10,485 (–35.0)	31,421 (21,650; 67,656)	28,680 (2796; 86,050)	–2740 (–8.7)
Nonzero costs, patient n	165	346	181 (109.7)	130	463	333 (256.2)	32	315	283 (884.4)
Mean ± SD	43,618 ± 44,151	48,942 ± 63,734	5324 (12.2)	52,510 ± 70,981	43,080 ± 66,177	–9430 (–18.0)	46,403 ± 34,640	62,588 ± 87,472	16,184 (34.9)
Median (Q1; Q3)	26,212 (14,990; 60,310)	24,660 (15,783; 57,039)	–1552 (–5.9)	29,916 (17,377; 53,143)	20,524 (5966; 52,693)	–9392 (–31.4)	31,421 (21,650; 67,656)	29,724 (3262; 86,370)	–1697 (–5.4)
**Outpatient visits**									
Total cost	165,229,755	182,955,168	17,725,413 (10.7)	62,832,431	80,468,457	17,636,026 (28.1)	24,675,886	29,818,339	5,142,453 (20.8)
Standard costs, patient n	15,362	15,183		2882	2878		1169	1162	
Mean ± SD	10,756 ± 26,701	12,050 ± 32,084	1294 (12.0)	21,802 ± 94,115	27,960 ± 93,624	6158 (28.2)	21,109 ± 100,596	25,661 ± 83,776	4553 (21.6)
Median (Q1; Q3)	4226 (1642; 10,315)	4598 (1763; 11,469)	372 (8.8)	6584 (2552; 17,830)	9307 (3257; 23,704)	2723 (41.4)	5090 (1719; 14,685)	9539 (4143; 21,553)	4449 (87.4)
Nonzero costs, patient n	15,361	15,183	–178 (–1.2)	2882	2877	–5 (–0.2)	1169	1162	–7 (–0.6)
Mean ± SD	10,756 ± 26,702	12,050 ± 32,084	1294 (12.0)	21,802 ± 94,115	27,970 ± 93,639	6168 (28.3)	21,109 ± 100,596	25,661 ± 83,776	4553 (21.6)
Median (Q1; Q3)	4227 (1642; 10,315)	4598 (1763; 11,469)	371 (8.8)	6584 (2552; 17,830)	9313 (3257; 23,704)	2729 (41.4)	5090 (1719; 14,685)	9539 (4143; 21,553)	4449 (87.4)
**Emergency department visits**									
Total cost	14,492,784	11,234,026	–3,258,759 (–22.5)	4,705,450	4,066,393	–639,057 (–13.6)	1,520,211	1,476,150	–44,060 (–2.9)
Standard costs, patient n	4368	3427		1168	968		407	361	
Mean ± SD	3318 ± 4780	3278 ± 5038	–40 (–1.2)	4029 ± 6268	4201 ± 5832	172 (4.3)	3735 ± 4241	4089 ± 12,625	354 (9.5)
Median (Q1; Q3)	2245 (1304; 3755)	2250 (1293; 3723)	4 (0.2)	2569 (1567; 4738)	2676 (1609; 4931)	107 (4.1)	2445 (1486; 4518)	2467 (1418; 4128)	21 (0.9)
Nonzero costs, patient n	4361	3423	–938 (–21.5)	1168	968	–200 (–17.1)	406	361	–45 (–11.1)
Mean ± SD	3323 ± 4782	3282 ± 5039	–41 (–1.2)	4029 ± 6268	4201 ± 5832	172 (4.3)	3744 ± 4242	4089 ± 12,625	345 (9.2)
Median (Q1; Q3)	2249 (1304; 3761)	2254 (1293; 3725)	5 (0.2)	2569 (1567; 4738)	2676 (1609; 4931)	107 (4.1)	2454 (1488; 4518)	2467 (1418; 4128)	12 (0.5)
**Prescription claims**									
Total cost	53,453,402	58,721,207	5,267,806 (9.9)	14,012,113	17,079,036	3,066,923 (21.9)	5,915,746	7,091,044	1,175,298 (19.9)
Standard costs, patient n	14,144	14,069		2604	2644		1097	1111	
Mean ± SD	3779 ± 15,333	4174 ± 16,026	395 (10.4)	5381 ± 13,865	6460 ± 15,983	1079 (20.0)	5393 ± 14,846	6383 ± 17,463	990 (18.4)
Median (Q1; Q3)	556 (162; 2340)	577 (166; 2799)	21 (3.8)	1362 (346; 5153)	1818 (445; 6091)	456 (33.5)	1096 (297; 4402)	1700 (406; 5822)	604 (55.1)
Nonzero costs, patient n	14,144	14,069	–75 (–0.5)	2604	2644	40 (1.5)	1097	1111	14 (1.3)
Mean ± SD	3779 ± 15,333	4174 ± 16,026	395 (10.4)	5381 ± 13,865	6460 ± 15,983	1079 (20.0)	5393 ± 14,846	6383 ± 17,463	990 (18.4)
Median (Q1; Q3)	556 (162; 2340)	577 (166; 2799)	21 (3.8)	1362 (346; 5153)	1818 (445; 6091)	456 (33.5)	1096 (297; 4402)	1700 (406; 5822)	604 (55.1)
**All medical costs (outpatient, inpatient, and prescription claims)**									
Total cost	273,689,317	312,850,265	39,160,948 (14.3)	114,396,043	146,365,442	31,969,399 (28.0)	38,344,378	65,396,345	27,051,967 (70.6)
Standard costs, patient n	15,416	15,313		2899	2890		1177	1172	
Mean ± SD	17,754 ± 40,000	20,430 ± 47,834	2677 (15.1)	39,461 ± 108,454	50,645 ± 113,091	11,185 (28.3)	32,578 ± 106,772	55,799 ± 113,215	23,221 (71.3)
Median (Q1; Q3)	6196 (2343; 16,739)	6717 (2497; 18,298)	521 (8.4)	13,543 (4388; 40,384)	16,906 (5460; 51,456)	3363 (24.8)	8493 (2978; 27,941)	18,578 (6643; 57,224)	10,085 (118.7)
Nonzero costs, patient n	15,416	15,313	–103 (–0.7)	2899	2889	–10 (–0.3)	1177	1171	–6 (–0.5)
Mean ± SD	17,754 ± 40,000	20,430 ± 47,834	2677 (15.1)	39,461 ± 108,454	50,645 ± 113,091	11,185 (28.3)	32,578 ± 106,772	55,799 ± 113,215	23,221 (71.3)
Median (Q1; Q3)	6196 (2343; 16,739)	6717 (2497; 18,298)	521 (8.4)	13,543 (4388; 40,384)	16,906 (5460; 51,456)	3363 (24.8)	8493 (2978; 27,941)	18,578 (6643; 57,224)	10,085 (118.7)

Standard cost patient n’s (used to calculate standard mean and median) reflect the number of patients who had any healthcare encounter for the specified outcome (eg, all patients with ≥ 1 outpatient visit during the specified time frame). Nonzero cost patient n’s (used to calculate nonzero mean and median) reflect the number of patients who had any costs associated with the specified outcome (eg, all patients with costs > 0 attributable to outpatient visits)

*COVID-19* coronavirus disease 2019, *ICU* intensive care unit

^a^The baseline phase was the 12 months before the index date, and the post-acute phase spanned from 1 to 13 months after the index date

^b^All costs are in US dollars rounded to the nearest dollar

Inpatient and outpatient costs also increased in the overall population and across all age and severity categories (Tables 8 and 9). Percentage increases in inpatient costs were driven primarily by cost increases among patients of any age who were admitted to the ICU during acute COVID-19 (+ 267.4%). Much of the increase in inpatient costs within this patient subpopulation was due to an 896% increase in 30-day all-cause readmissions (Table 7), resulting in a 1227.7% cost escalation during the post-acute phase. Outpatient costs increased more modestly and were greatest among older patients and those who were hospitalized (with or without ICU admission) during acute COVID-19. Overall outpatient cost increases were observed despite decreases associated with ED visits across all age and severity categories.

## Discussion

In this retrospective analysis encompassing > 2 years of healthcare data from nearly 20,000 high-risk individuals diagnosed with COVID-19, resource use and costs were substantially higher in the year after acute COVID-19 illness compared with the previous year. Consistent with previous observations [15], increases were greatest among older individuals and those who required hospitalization for acute COVID-19; however, notable changes were observed even among younger patients and those who had less severe acute disease.

We found increases in incidence of blood-related, neurologic, and psychiatric disorders, all of which are consistent with previous reports on post-COVID conditions [32–34]. We observed a 16.3% increase in the ICD-10 diagnostic codes comprising “diseases of the blood and blood-forming organs” along with 31.6% and 29.9% increases in blood factor prescriptions and hemostatic modifier prescriptions, respectively. This is consistent with the potential for COVID-19 to cause persistent changes in the mechanisms underlying coagulation and hemostasis [33]. An 11.1% increase in “diseases of the nervous system” and a 7.7% increase in “mental and behavioral disorders” were also observed alongside increased prescribing of neurologic/neuromuscular disorder treatments and psychotherapeutic drugs (+ 12.7% and + 11.1%, respectively). The magnitude of increases in mental health–related prescriptions were relatively similar among patients who were not hospitalized during acute COVID-19 illness compared with the overall cohort, supporting previous reports that identified long-term impairments in mood, anxiety, and cognitive functioning that were unrelated to COVID-19 severity or hospitalization [32, 34, 35]. Our results are further corroborated by a longitudinal study of UK Biobank participants, in which cognitive declines observed > 3 months after COVID-19 diagnosis were significant even among nonhospitalized cases and were associated with structural changes in the brain [35]. In contrast with recent CDC data [10], we did not observe an overall increase in respiratory conditions because acute respiratory infections decreased. CDC defined respiratory conditions only as acute pulmonary embolism, asthma, or respiratory symptoms.

The percentages of outpatient and inpatient medical service use were substantially higher during the post-acute phase compared with baseline, including a 165.6% increase in total days spent in the hospital and a 249.9% increase in 30-day all-cause readmissions. This increase in healthcare utilization was directly reflected in observed medical costs. Total costs increased by 23.0% in the overall population; increases were greater among patients aged ≥ 65 years compared with the younger population and ranged from 14.3% among patients who were not hospitalized for acute COVID-19 to 70.6% among those admitted to the ICU.

Mean overall per-patient medical cost during the year after the acute COVID-19 phase was approximately $27,000 (whereas the baseline cost was approximately $22,000). Although there are no direct comparisons that can be made with previous studies, cost estimates of post-COVID conditions based on similar diagnoses (eg, myalgic encephalomyelitis/chronic fatigue syndrome) were nearly $9000 per person per year [36]. The higher values observed in our study may reflect a greater impact of COVID-19 than similar conditions but could also be a result of our high-risk patient population. Notably, studies comparing post-COVID conditions with long-term sequelae after seasonal influenza found a greater symptom, diagnosis, and healthcare resource burden with COVID [12, 24].

Substantial decreases were observed in the percentages of acute upper and lower respiratory infections, prescriptions for cough/cold/flu preparations, and ED visits. These data are consistent with early reports in the United States that show a sharp decline in both influenza rates and ED visits after the onset of the pandemic, including ED visits specifically for non-COVID upper respiratory infections [37–39]. This decline was likely due to a combination of reduced influenza circulation because of COVID-19 mitigation strategies and the reluctance of patients to seek treatment because of the perceived risk of contracting COVID-19 in a healthcare setting [37, 39].

A main strength of this study was that all patients served as their own control, which inherently adjusts for potential confounders, including patient demographics and stable characteristics, such as healthcare-seeking behavior. Our study had some limitations, including that the population was limited to commercially insured individuals who were diagnosed early in the pandemic and who survived the acute phase of COVID-19. There was also potential for incomplete data capture (due to nonbillable diagnoses) and surveillance bias by which those who contracted COVID-19 were under higher medical scrutiny following their diagnosis. It is important to note that the study was conducted during a time of low preexisting immunity (ie, before vaccination and previous infection) and before the emergence of and SARS-CoV-2 variants of concern. Such factors could limit the generalizability of the findings to the current landscape. Additionally, baseline assessments were performed during the pre-pandemic period, whereas post-acute COVID-19 assessments were performed during a public health emergency that altered healthcare practices, access, and considerations; these changes may have affected clinical burden and healthcare costs during the post-acute COVID-19 period. Because this study included only high-risk individuals, a companion report describes results from a separate cohort of patients without high risk of developing severe COVID-19.

## Conclusion

The health and economic burden of post-COVID conditions among high-risk US adults is substantial. Although the greatest impacts were observed among patients aged ≥ 65 years and those who were admitted to the ICU for acute COVID-19, increases in most outcomes were apparent even among younger individuals and those who did not require COVID-19 hospitalization. These results improve our understanding of post-COVID conditions and associated costs, as well as support hypothesis generation for future work characterizing the COVID-19 impact on individuals and society.

### Supplementary Information

**Additional file 1: Table S1.** Reasons for exclusion from the study.

**Additional file 2: Table S2.** Diagnoses of the respiratory system during the baseline and post-acute phases in the overall population (*N*=19,558)^a^.

**Additional file 3: Figure S1.** Percentage change from the baseline phase to the post-acute phase in frequency of ICD-10-CM “diseases of the respiratory system” in the overall population (*N*=19,558). Diagnosis codes shown include those applicable to ≥2% of the baseline population.

**Additional file 4: Table S3.** Medication prescriptions during the baseline and post-acute phases in the overall population (*N*=19,558)^a^.

**Additional file 5: Table S4.** Hospital discharge status during baseline and post-acute phases^a^ in the overall population and stratified by age.

## Data Availability

Upon request, and subject to review, Pfizer will provide the data that support the findings of this study. Subject to certain criteria, conditions, and exceptions, Pfizer may also provide access to the related individual de-identified participant data. See https://www.pfizer.com/science/clinical-trials/trial-data-and-results for more information.

## Abbreviations

CDC: US Centers for Disease Control and Prevention
CDM: Optum’s de-identified Clinformatics® Data Mart Database
CPT: Current Procedural Terminology
ED: Emergency department
HCPCS: Healthcare Common Procedure Coding System
ICD-10: International Classification of Diseases, 10th Revision
ICD-10-CM: International Classification of Diseases, 10th Revision, Clinical Modification
ICD-10-PCS: International Classification of Diseases, 10th Revision, Procedure Coding System
ICU: Intensive care unit
LOS: Length of stay
LTCF: Long-term care facility
NC: Not calculable
NDC: National Drug Code
PASC: Post-acute sequelae of COVID-19
Q1: Quartile 1
Q3: Quartile 3
SAS: Statistical analysis software
SNF: Skilled nursing facility
USC: Uniform System of Classification
USD: United States dollars
