# Supplementary material for: Substantial health and economic burden of COVID-19 during the year after acute illness among US adults at high risk of severe COVID-19
Source: BMC Med. 2024 Feb 1;22:46. doi: 10.1186/s12916-023-03234-6 (PMC10836000; doi:10.1186/s12916-023-03234-6)
Supplement: Supplementary file 1 — Additional file 1: Table S1. Reasons for exclusion from the study. [file 12916_2023_3234_MOESM1_ESM.pdf]

Additional Files

Table S1. Reasons for Exclusion From the Study

Cohort Selection Step	N	%
All patients enrolled in database during April 2020–May 2020	16,909,650	100
and with ≥1 ICD-10 diagnosis of confirmed COVID-19 in any position	70,924	0.419
and exclude patients with an ICD-10 code for confirmed COVID-19 (U07.1) before April 1, 2020 (preindex)	68,297	0.404
and with continuous enrollment 12 months (360 days) before the index date (45-day gap)	50,463	0.298
and with continuous enrollment 13 months (390 days) after the index date (45-day gap)	32,551	0.192
and aged ≥18 at the index date	32,054	0.190
and without any LTCF/SNF/inpatient rehab/hospice claim before or at the index date	23,458	0.139
and exclude patients admitted earlier than 5 days before IP index COVID-19 diagnosis	23,392	0.138
and alive in the data/no death[a] code at 30 days after the COVID-19 diagnosis date (index date)	23,350	0.138
and has ≥1 characteristic or condition (within 12 months before COVID-19 diagnosis - excluding index date) associated with higher risk for severe COVID-19	19,558	0.116

ICD-10: International Classification of Diseases, 10th Revision; LTCF=long-term care facility, SNF=skilled nursing facility.

[a]There is no accurate date of death available, only month and year of death, so it was approximated to the last day of the month.
