# Supplementary material for: Substantial health and economic burden of COVID-19 during the year after acute illness among US adults at high risk of severe COVID-19
Source: BMC Med. 2024 Feb 1;22:46. doi: 10.1186/s12916-023-03234-6 (PMC10836000; doi:10.1186/s12916-023-03234-6)
Supplement: Supplementary file 2 — Additional file 2: Table S2. Diagnoses of the respiratory system during the baseline and post-acute phases in the overall population (N=19,558)a. [file 12916_2023_3234_MOESM2_ESM.pdf]

Table S2. Diagnoses of the Respiratory System During the Baseline and Post-Acute Phases in the Overall Population (N=19,558)[a]

ICD-10-CM Diagnosis Description	ICD-10-CM Diagnosis Code	Baseline Phase, n (%)	Post-Acute Phase, n (%)	Change From Baseline to Post-Acute Phase, Δ (% Change)
Other respiratory diseases principally affecting the interstitium	J80–J84	562 (2.9)	898 (4.6)	336 (59.8)
Other diseases of pleura	J90–J94	484 (2.5)	695 (3.6)	211 (43.6)
Other diseases of the respiratory system	J95–J99	1873 (9.6)	2447 (12.5)	574 (30.6)
Influenza and pneumonia	J09–J18	2325 (11.9)	2431 (12.4)	106 (4.6)
Chronic lower respiratory diseases	J40–J47	4178 (21.4)	4056 (20.7)	–122 (–2.9)
Other diseases of upper respiratory tract	J30–J39	3591 (18.4)	3201 (16.4)	–390 (–10.9)
Acute upper respiratory infections	J00–J06	5926 (30.3)	2569 (13.1)	–3357 (–56.6)
Other acute lower respiratory infections	J20–J22	1918 (9.8)	589 (3.0)	–1329 (–69.3)

ICD-10-CM, International Classification of Diseases, Tenth Revision, *Clinical Modification*.
[a]The baseline phase was the 12 months before the index date, and the post-acute phase spanned from 1 to 13 months after the index date
