# Supplementary material for: Substantial health and economic burden of COVID-19 during the year after acute illness among US adults at high risk of severe COVID-19
Source: BMC Med. 2024 Feb 1;22:46. doi: 10.1186/s12916-023-03234-6 (PMC10836000; doi:10.1186/s12916-023-03234-6)

Figure S1. Percentage change from the baseline phase to the post-acute phase in frequency of ICD-10-CM “diseases of the respiratory system” in the overall population (N=19,558). Diagnosis codes shown include those applicable to ≥2% of the baseline population. ICD-10, International Classification of Diseases, Tenth Revision*, Clinical Modification*.

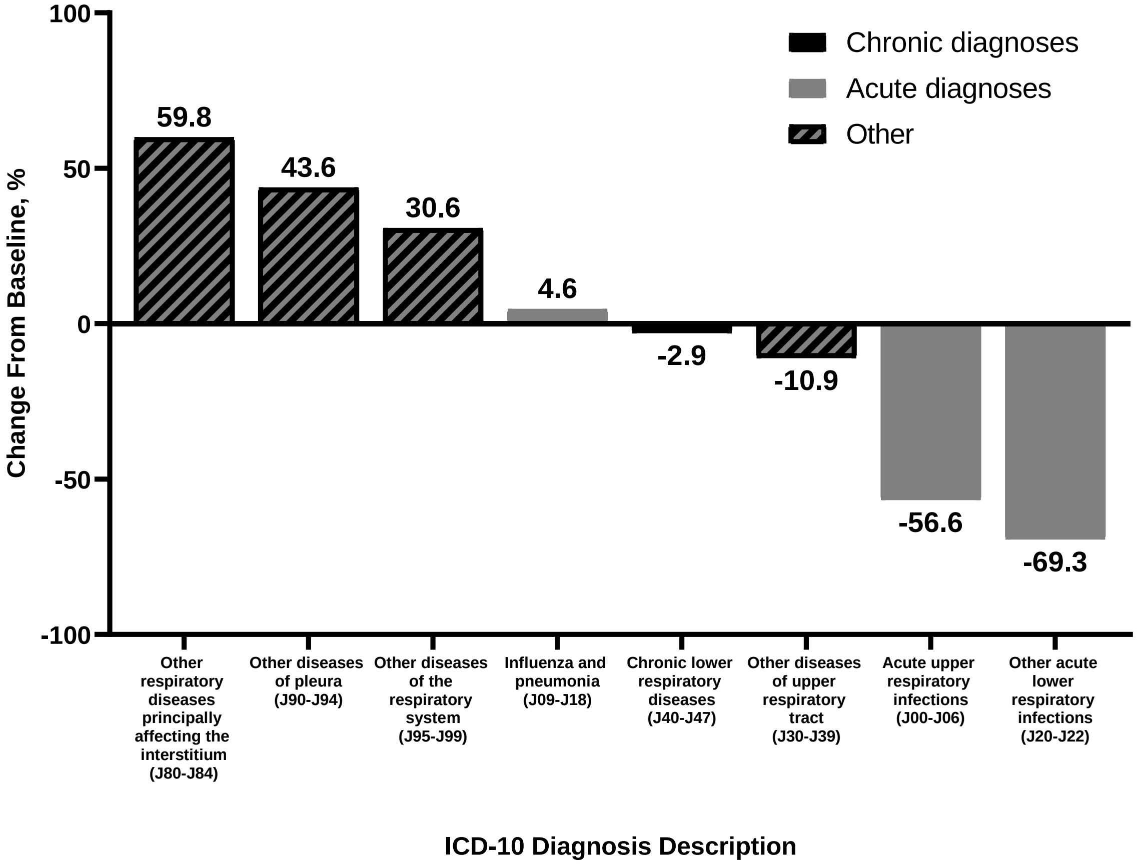

Supplement: Supplementary file 3 — Additional file 3: Figure S1. Percentage change from the baseline phase to the post-acute phase in frequency of ICD-10-CM “diseases of the respiratory system” in the overall population (N=19,558). Diagnosis codes shown include those applicable to ≥2% of the baseline population. [file 12916_2023_3234_MOESM3_ESM.pdf]
