# Supplementary material for: Substantial health and economic burden of COVID-19 during the year after acute illness among US adults at high risk of severe COVID-19
Source: BMC Med. 2024 Feb 1;22:46. doi: 10.1186/s12916-023-03234-6 (PMC10836000; doi:10.1186/s12916-023-03234-6)
Supplement: Supplementary file 4 — Additional file 4: Table S3. Medication prescriptions during the baseline and post-acute phases in the overall population (N=19,558)a. [file 12916_2023_3234_MOESM4_ESM.pdf]

Table S3. Medication Prescriptions During the Baseline and Post-Acute Phases in the Overall Population (N=19,558)[a]

USC Medication Class Description	Baseline Phase, n (%)	Post-Acute Phase, n (%)	Change From Baseline to Post-Acute Phase, Δ (% Change)
Vitamins	771 (3.9)	1137 (5.8)	366 (47.5)
Miscellaneous preparations	831 (4.2)	1147 (5.9)	316 (38.0)
Blood factors	675 (3.5)	888 (4.5)	213 (31.6)
Hemostatic modifiers	2093 (10.7)	2719 (13.9)	626 (29.9)
Nutrients & supplements	980 (5.0)	1246 (6.4)	266 (27.1)
Cardiac agents	1203 (6.2)	1485 (7.6)	282 (23.4)
Antineoplastic targeted therapy	536 (2.7)	644 (3.3)	108 (20.1)
Thyroid therapy	2222 (11.4)	2553 (13.1)	331 (14.9)
Neurologic/neuromuscular disorders	3790 (19.4)	4271 (21.8)	481 (12.7)
Gastrointestinal	5002 (25.6)	5595 (28.6)	593 (11.9)
Psychotherapeutic drugs	5876 (30.0)	6530 (33.4)	654 (11.1)
Diagnostic aids	3288 (16.8)	3652 (18.7)	364 (11.1)
Genitourinary	3352 (17.1)	3668 (18.8)	316 (9.4)
Antihyperlipidemic agents	7831 (40.0)	8510 (43.5)	679 (8.7)
Diabetes therapy	4264 (21.8)	4623 (23.6)	359 (8.4)
Antiseptics	669 (3.4)	723 (3.7)	54 (8.1)
Diuretics and aquaretics	3843 (19.6)	4144 (21.2)	301 (7.8)
Hospital solutions	883 (4.5)	950 (4.9)	67 (7.6)
Laxatives	1150 (5.9)	1236 (6.3)	86 (7.5)
Vascular agents	9800 (50.1)	10,361 (53.0)	561 (5.7)
Nondrug products	713 (3.6)	753 (3.9)	40 (5.6)
Musculoskeletal	2817 (14.4)	2949 (15.1)	132 (4.7)
Dermatologicals	1544 (7.9)	1595 (8.2)	51 (3.3)
Contraceptives	649 (3.3)	670 (3.4)	21 (3.2)

Antifungal agents	2530 (12.9)	2610 (13.3)	80 (3.2)
Anesthetics	1129 (5.8)	1160 (5.9)	31 (2.7)
Sedatives & hypnotics	894 (4.6)	910 (4.7)	16 (1.8)
Amebicide/trichomonacides/antibacterials	596 (3.0)	606 (3.1)	10 (1.7)
Analgesics	5083 (26.0)	5140 (26.3)	57 (1.1)
Antiarthritics	5321 (27.2)	5361 (27.4)	40 (0.8)
Ophthalmic preparations	2735 (14.0)	2701 (13.8)	–34 (–1.2)
Hormones	7814 (40.0)	6965 (35.6)	–849 (–10.9)
Allergy/cold preparations	960 (4.9)	850 (4.3)	–110 (–11.5)
Antinauseants	2670 (13.7)	2328 (11.9)	–342 (–12.8)
Respiratory therapy	5889 (30.1)	5033 (25.7)	–856 (–14.5)
Anti-infectives, systemic	10,284 (52.6)	7857 (40.2)	–2427 (–23.6)
Antivirals	1994 (10.2)	1188 (6.1)	–806 (–40.4)
Antimalarials	527 (2.7)	240 (1.2)	–287 (–54.5)
Cough/cold/flu preparations	1929 (9.9)	508 (2.6)	–1421 (–73.7)

USC, Uniform System of Classification.
[a]The baseline phase was the 12 months before the index date, and the post-acute phase spanned from 1 to 13 months after the index date.
