# Supplementary material for: Substantial health and economic burden of COVID-19 during the year after acute illness among US adults at high risk of severe COVID-19
Source: BMC Med. 2024 Feb 1;22:46. doi: 10.1186/s12916-023-03234-6 (PMC10836000; doi:10.1186/s12916-023-03234-6)
Supplement: Supplementary file 5 — Additional file 5: Table S4. Hospital discharge status during baseline and post-acute phasesa in the overall population and stratified by age. [file 12916_2023_3234_MOESM5_ESM.pdf]

Table S4. Hospital Discharge Status During Baseline and Post-Acute Phases[a] in the Overall Population and Stratified by Age

	All Patients (N=19,558)			Patients Aged 18–64 Years (n=9381)			Patients Aged ≥65 Years (n=10,177)		
	**Baseline Phase**	**Post-Acute Phase**	**Change From Baseline to Post-Acute Phase, Δ (% Change)**	**Baseline Phase**	**Post-Acute Phase**	**Change From Baseline to Post-Acute Phase, Δ (% Change)**	**Baseline Phase**	**Post-Acute Phase**	**Change From Baseline to Post-Acute Phase, Δ (% Change)**
Hospitalizations, n	3642	5301		1314	1528		2328	3773	
Discharge status, n (%)									
Discharged to home or self-care	2589 (71.1)	2529 (47.7)	–60 (–2.3)	1037 (78.9)	1019 (66.7)	–18 (–1.7)	1552 (66.7)	1510 (40.0)	–42 (–2.7)
Discharged to home under care of home health service organization	711 (19.5)	1106 (20.9)	395 (55.6)	108 (8.2)	169 (11.1)	61 (56.5)	603 (25.9)	937 (24.8)	334 (55.4)
Discharged to other facility[b]	202 (5.5)	985 (18.6)	783 (387.6)	61 (4.6)	159 (10.4)	98 (160.7)	141 (6.1)	826 (21.9)	685 (485.8)
Left against medical advice or discontinued care	48 (1.3)	75 (1.4)	27 (56.3)	31 (2.4)	34 (2.2)	3 (9.7)	17 (0.7)	41 (1.1)	24 (141.2)
Still patient/transferred within institution	8 (0.2)	451 (8.5)	443 (5538)	5 (0.4)	58 (3.8)	53 (1060)	3 (0.1)	393 (10.4)	390 (13,000.0)
Unknown status	84 (2.3)	155 (2.9)	71 (84.5)	72 (5.5)	89 (5.8)	17 (23.6)	12 (0.5)	66 (1.7)	54 (450.0)

Percentages were calculated in relation to the total number of hospital discharges within the cohort during the specified time frame.
[a]The baseline phase was the 12 months before the index date, and the post-acute phase spanned from 1 to 13 months after the index date.
[b]Includes short-term general hospital, skilled nursing facility, intermediate care facility, federal healthcare facility, home hospice, medical facility hospice, inpatient rehabilitation facility, long-term care hospital, nursing facility certified under Medicare, psychiatric hospital or psychiatric distinct part/unit of a hospital, critical access hospital, or other type of healthcare institution.
